# Integration of deworming into primary healthcare in Western Kenya: a quasi-experimental evaluation

**DOI:** 10.3389/fpubh.2026.1859409

**Published:** 2026-07-10

**Authors:** Josphat Martin Muchangi, Wyckliff Omondi, Meshack Ndirangu, Carol Karutu, Irene Chami, Gilbert Wangalwa, Michael Ofire, Robert Ofwete, Kioko Kithuki, Samuel Kamau, Nashon Kaloki, Lenson Kariuki, Paul Kibati, Japheth Athanasio, Geoffrey Njuhi, Yvonne Opanga, Emmanuel Musombi, Chitiavi Juma, Dollycate N. Wanja, Festus Kipkorir, Hesbon Aoko

**Affiliations:** 1 Amref Health Africa, Nairobi, Kenya; 2 Vector-Borne & Neglected Tropical Diseases Unit, Ministry of Health, Nairobi, Kenya; 3 The END Fund, Nairobi, Kenya; 4 Reinit Research Limited, Nairobi, Kenya; 5 Department of Health Systems Strengthening, Ministry of Health, Nairobi, Kenya; 6 Children Investment Fund Foundation, Nairobi, Kenya

**Keywords:** coverage, deworming, integration, Kenya, mass drug administration, neglected tropical disease, primary healthcare

## Abstract

**Introduction:**

The WHO 2030 NTD roadmap calls for integrating mass drug administration (MDA) into primary healthcare (PHC) systems. A coverage evaluation survey conducted in Western Kenya exposed the persistent limitations of mass drug administration campaigns, showing that nearly 25% of surveyed individuals never received treatment. The effect of stock-outs and ineffective mobilizations were also noted. To consolidate the gains from previous mass drug administrations (MDAs), integration of MDA into Primary Health Care (PHC) services was piloted through the Deworming Innovation Fund by leveraging community health structures and digital health platforms for improved delivery.

**Methodology:**

A quasi-experimental controlled before-and-after (CBA) study was conducted across 24 wards that were selected based on high helminth prevalence, comparable population size, facility availability, and accessibility for monitoring (12 intervention, 12 control) in Bungoma, Kakamega, Vihiga, and Trans-Nzoia counties. The start date of intervention was in April 2025, *n* = 1,229) and the end date of endline data collection was in June 2025, *n* = 1,130). The intervention took 3 months. Household surveys measured preventive chemotherapy (PC) coverage, epidemiological coverage, awareness sources, and treatment uptake. Mixed methods included key informant interviews and focus group discussions. The primary outcome was PC coverage (target ≥75% per WHO).

**Findings:**

The integrated PHC model in the intervention group helped to increase deworming coverage from 58.4% to 75%; representing an adjusted odd ratio of 2.14 (95% CI: 1.48–3.09). This is in comparison to other control areas where coverage changed only marginally from 57.1% to 60.3%. However, the possible unmeasured programme and confounding implementation limits the quasi-experimental design. As a result, results are mostly relevant to county healthcare managers, NGOs and policymakers. These are people seeking scalable child healthcare interventions in endemic environments. Health facilities became a prominent awareness source only in intervention sites (30% at endline vs. 15% at baseline). In contrast, our study found lower non-treatment rates at 5% (compared to about a quarter reported in the 2024 MDA Coverage Evaluation Survey (CES), potentially reflecting the impact of the integrated approach or differences in recall period and sampling. The 5% of households reporting never receiving treatment were associated with various reasons, including; ineligibility (56%) or absence (17%), not lack of awareness (1%). Control wards showed sharp declines in awareness post-campaign, while intervention wards maintained or improved awareness through routine facility contacts.

**Recommendations:**

PHC-integrated deworming achieved effective coverage comparable to traditional campaigns, with superior sustainability of awareness.

## 1 Introduction

The global health community is now committed tackling the burden of Neglected Tropical Diseases (NTDs), as increased attention has been directed toward this set of diseases of poverty. This is best seen in the recognition of the need reduce NTD morbidity and mortality within the Sustainable Development Goal (SDG) 3.3 (1) and the World Health Organization NTD roadmap 2021–2030 (2). The study hypothesized that PHC-integrated deworming would achieve non-inferior preventive chemotherapy coverage (≥75%) compared to campaign-based MDA, while improving awareness sustainability. In the intervention package, there was routine deworming into PHC through healthcare worker and CHVs, digital child registers, facility-based blood screening, community mobilization campaigns, and strengthened drug supply chains. The primary outcome (PC coverage ≥75% per WHO) was recorded.

Other events, such as the London Declaration that brought together key stakeholders into a commitment to end at least 10 of the diseases by 2020, set the pace for action. The World Health Organization (WHO) regional strategic plans for Africa and Asia identified preventive chemotherapy as part of the intervention packages for the elimination efforts (3). Mass drug administration (MDA) campaigns, established through public-private partnerships are designed to deliver preventive chemotherapy drugs to populations at risk (4). This strategy specifically targets populations at risk, especially school age children. As a result, the literature shows great progress in the last 20 years, such as a 26% reduction in the number of people requiring interventions against NTDs (5). Additional literature also reports a 29% reduction in the number of NTD cases between 1990 and 2021 (6).

MDAs are mostly run separately from the established health care structures in the targeted population. Emphasis on this vertical methodology has led to reduced attention to other forms of neglect such as weak health systems in endemic regions, and it is getting clear that MDAs alone will not be sufficient for elimination. In spite of the progress made against helminth infections, we still have a long way to go. In 2022, the were 26% less people needing interventions as compare to 2010, but this is not sufficient to enable us to meet the Global NTD Roadmap target of a 90% reduction by 2030 (7). There are unmet needs that affect both areas where MDAs have succeeded and where they have not. Stopping MDAs after reducing the disease burden, or successfully intensifying the campaigns for elimination may mean the redirection of both domestic and international funds to more pressing health issues. This would potentially open a window for resurgence of prevalence to pre-MDA levels (8, 9). Progress in achieving the targets set calls for the integration of control activities into the broader health system, rather than running them in parallel. The 2030 NTD global roadmap actually calls for the integration of efforts both across NTDs and within health systems (10). We must ensure that the whole pipeline, from detection to treatment, are embedded within the primary healthcare systems (11).

Kenya has conducted Mass Drug Administration (MDA) as a national strategy to eliminate and control NTDs, especially schistosomiasis (bilharzia) and soil-transmitted helminths. Kenya has the National Master Plan for the Elimination of NTDs (2023 to 2027), which mirrors the WHO NTD road map 2021 to 2030 (12). Due to these MDAs, work infestation prevalence has significantly reduced from 32% to 5.8% from 2012 to 2022, showing an 80%+ reduction in prevalence.

Kenya's PHC system, as outlined in the Primary Health Care Act (2023) and operationalized through the Kenya Community Health Policy (2020–2030) and Primary Health Care Networks Guidelines, follows a hub-and-spoke model with community health units, dispensaries, health centers, sub-county, and county referral hospitals forming an integrated referral network. The electronic Community Health Information System (eCHIS) supports real-time tracking of patient services at the community level, ensuring efficient forward and reverse referral between communities and health facilities. The recent transition to the Social Health Authority (SHA) in October 2024 further strengthens opportunities for scaling up integrated NTD interventions within UHC initiatives. NTDs, including STH and SCH cases requiring clinical management, are already part of the SHA benefits package. By consolidating financing and service delivery under a single authority, SHA provides a strong platform for embedding and sustainably financing routine diagnosis, treatment, and prevention of NTDs within the essential health benefits package.

This institutionalization is directly aligned with the Kenya National NTD Master Plan (2023–2027) and the WHO NTD Roadmap (2021–2030). Both frameworks emphasize integration of preventive chemotherapy into PHC and UHC platforms to ensure sustainable delivery, achievement of the 75% minimum MDA coverage threshold across all eligible populations, strengthened domestic financing to reduce reliance on donor-driven vertical campaigns, and enhanced monitoring systems to track coverage and equity in access to NTD services.

This study directly addresses critical evidence gaps that remain despite existing research. For example, earlier work such as Pullan et al. (13) highlighted the burden and risk factors for STH and SCH in Kenya but did not explore the comparative effectiveness of delivery models or assess the feasibility of integration into PHC systems. By contrast, this study not only provides comparative data between PHC-integrated and campaign-based delivery models but also explores health system readiness, county-level budgeting processes, and operational strategies that can inform scale-up within the SHA and UHC reforms. Therefore, in this way, the study contributes both to Kenya's NTD elimination agenda and to the global evidence base on sustainable, system-anchored approaches to ending STH and SCH as public health problems by 2030 (5).

Western Kenya has historically carried a high burden of Soil-Transmitted Helminths (STH) and Schistosomiasis (SCH) due to environmental and socio-economic conditions that favor transmission. However, recent evidence shows progress. Precision mapping studies conducted in 2021 under the Deworming Innovation Fund (14) revealed localized heterogeneity in prevalence across Bungoma, Kakamega, Vihiga, and Trans-Nzoia counties. This study yielded information to refine treatment targeting and exposed challenges related to community acceptance, logistics, and health system capacity. More recently, a 2024 MDA Coverage Evaluation Survey (CES) highlighted persistent gaps in program delivery. While compliance among those reached was high, only 60% of wards achieved the minimum 75% preventive chemotherapy threshold. The CES further revealed that nearly one-quarter of surveyed individuals had never received STH treatment, mainly due to absence during MDA, not being offered drugs, or lack of awareness. Stock-outs of praziquantel, and inconsistent social mobilization, especially for SCH, also limited MDA impact.

To consolidate the gains of MDAs in the region, and address ongoing challenges, integration of MDA into PHC services was piloted in Western Kenya through the Deworming Innovation Fund. This study evaluated the integration intervention, which focused on strengthening the service delivery, sustainability, recording and reporting of STH and SCH treatment activities. The primary objective of the study was to evaluate the effectiveness of PHC-integrated deworming delivery compared with the conventional campaign-based MDA approach in achieving program reach, preventive chemotherapy coverage, compliance, and epidemiological coverage. The study comprised of two phases: a baseline and endline evaluation. The intervention phase implemented the intervention package in selected wards within the four counties for 3 month and was then evaluated for coverage and effectiveness. Data collected at baseline was used to compare outcomes for both arms of the study. This paper presents the results of the evaluation and gives recommendations for future integration activities in the Kenyan context.

## 2 Materials and methods

### 2.1 Study design

This study employed a quasi-experimental controlled before-and-after (CBA) design, implemented as a non-randomized community trial. The study was conducted in four counties of Western Kenya, where previous MDA campaigns have been conducted and both therapeutic coverage and coverage evaluation survey data have been reported.

A baseline assessment was conducted in April 2025 to acquire reference data on treatment coverage, service delivery models, community perceptions, and health system readiness across intervention and control wards. The control group consisted of health facilities and communities following the traditional MDA approach, while the intervention group received treatment through PHC-integrated delivery channels. The endline evaluation employed mixed methods, combining quantitative surveys with qualitative approaches to capture both outcome measures and contextual insights. Qualitative interviews and quantitative survey findings were integrated through triangulation and convergence, comparing results across methods to validate patterns, explain inconsistencies, and provide complementary contextual insights. Data collection took place in the same counties and wards assessed during the baseline to ensure comparability. Outcomes were contrasted between intervention sites, where treatment was integrated into PHC services, and control sites, where the conventional MDA campaign model continued.

### 2.2 Study area and population

The baseline and endline assessments were conducted in the same sites, covering the counties of Vihiga, Kakamega, Bungoma, and Trans Nzoia. Randomization was not applied, as site selection was based on programmatic feasibility and the capacity of local health systems. In particular, The END Fund's Subnational Interruption of Transmission of STH/SCH Project was conducted in these counties. This meant that both therapeutic and coverage evaluation survey data for previous MDA campaigns were available. Altogether, the study covered 15 sub-counties, 24 wards, 72 villages, and 24 health facilities, distributed across both control (campaign-based delivery) and intervention (PHC-integrated delivery) arms.

The study population included all individuals eligible for treatment in the targeted geographical regions of Western Kenya. In line with the national guidelines and the baseline assessment framework, the survey population comprised:

a) All eligible community members, including school-age children (SAC; 5–14 years) and adults (15 years and above), who were targeted for SCH treatment at baseline and continued to be assessed at endline.
b) Pre-school-age children (PSAC; 1–4 years), school-age children (SAC; 5–14 years), and adults (15 years and above) who were included in the baseline survey and remained eligible for targeted STH treatment.

To enhance internal validity and ensure geographical balance, randomization was stratified by county to account for contextual variations in health system capacity, disease burden, and population dynamics. Participants who consented to participating for the baseline assessment were followed up for the endline assessment.

### 2.3 Intervention package

The intervention group received SCH/STH treatment integrated into PHC through multiple service delivery channels including:

a) The administration of SCH/STH treatment by health workers at health facilities, where deworming was incorporated into routine child health services, antenatal care visits, and school health programs. Therefore, all eligible children and women had access to treatment during scheduled visits.
b) The SCH/STH treatment was given to both the intervention and control group at least once during the 3 months.
c) Community health workers partnered with schools and community-based programs for mass deworming campaigns.
d) SCH/STH drug supplies were integrated into routine PHC stock management systems to ensure timely replenishment through county health supply chains. This included the implementation of Electronic Community Health Information System to enable the tracking of drug availability, distribution patterns, and consumption rates to avoid stockouts.
e) The Community Health Promoters (CHPs) were engaged to facilitate awareness, mobilization, and follow-up activities during their routine community health visits. They were trained to conduct household and school-based sensitization sessions to dispel myths and misconceptions on deworming, encourage treatment uptake, and ensureadherence.
f) Health workforce capacity building through targeted training programs, mentorship and on-the-job coaching on treatment delivery, surveillance, adverse event monitoring, patient counseling, data collection, and reporting.
g) Advocacy and behavioral change through targeted health education campaigns integrated into existing community outreach activities such as immunization days, school health programs, and Malezi Bora initiatives for sustained awareness and community engagements.
h) Data fidelity was measured through conducting routine verification of all completed tools, doing consistency checks between survey tools and registers, and conducting pre-interviews for a small sample of the participants.
Supervisory checklists, stock availability reports, and attendance logs were used in doing implementation quality and monitoring.
j) Monthly review meetings were conducted to identify any gaps in delivery, resolve any challenges in operations and maintain standardized implementation across all wards that were taking part in the study.

### 2.4 Data collection and analysis methods

The study employed a mixed methods approach, combining both qualitative and quantitative methods for both outcome measures and contextual insights. Mixed methods approach was preferred as it enabled integration of the findings, through triangulation of the primary and secondary findings. Mixed methods approach allowed the researchers to collect quantitative information through questionnaires and health facility assessments, while qualitative information was through key informant interviews and focus group discussions. Baseline data were collected in April 2025, and endline data in June 2025 which was 3 months after the start of the intervention.

#### 2.4.1 Quantitative methods

Quantitative data were collected through household surveys and health facility assessments. Structured questionnaires were administered to households in both intervention and control wards to capture information (variables) on program reach (communication channels, MDA awareness and uptake, and delivery of STH/SCH treatment, and STH/SCH deworming program reach), preventive chemotherapy coverage (chemo coverage, compliance with STH/SCH deworming), and epidemiological coverage (epidemiological coverage for STH/SCH deworming, effectiveness of STH.SCH deworming, and “never treated” households). The variables were measured using Yes and No options, and open-ended questions.

Facility-level structured interviews were also used to assess service readiness, human resource capacity, and the extent of integration of SCH/STH into PHC, stock availability, and reporting practices. About 24 health facilities were assessed and service readiness domains included the capacity of staff, availability of drugs, reporting tools, equipment, and infection-control supplies. Trained research assistants were used in conducting assessments with supervision from field coordinators. A total of 508 participants were selected for the intervention group, and a similar number for control, leading to a total of 1,016 participants. The sample size was calculated using Cochran (21) sample size formular;


$$\begin{array}{l} n=\frac{\left(Z_{\alpha /2}\;p\left(1-p\right)\right)}{d^{2},} \end{array}$$

where *p* was expected MDA coverage, taken at 75% (0.75) based on previous studies, and *d* as the margin of error, at 5% (0.05), with a *Z*_α/2_ of 1.96 for a 95% confidence level. Further, there were adjustments for design, and for non-response rates.

Sampling for households and health facilities was GIS-centric, to enable the research assistants in tracing the same households and health facilities at end line, using the GPS coordinates obtained at baseline. The retention rate stood at 92.0%. Attrition was mainly because of migration, relocation of household, refusal to take part, and temporary absence of eligible respondents. The multistage stratified sampling approach was used in selecting households. Wards were first stratified by helminth prevalence and then villages selected randomly within every ward. The sampling entailed updated lists of households obtained from CHVs and village administrators. Inclusion criteria included those households that had at least one child of 5–14 years and adults who are 15 years and above and residing in the areas for at least 6 months. Households were excluded if residents were non-residential, vacant, or if no eligible child or adult was present.

Analysis was done using Stata version 18 for generating descriptive statistics (means, frequencies, and proportions), chi-square tests and logistic regression. Chi-square tests were done to test associations and differences between samples. In logistic regression, binary variables were created, for instance, aware and not aware of the MDA and MDA integrated into PHC and MDA not integrated into PHC. The variables were coded into categorical options, enabling logistic regression. Presentation of data was in tables.

#### 2.4.2 Qualitative methods

Qualitative data were gathered through 18 key informant interviews (KIIs) and 12 focus group discussions (FGDs). KIIs were conducted with county and sub-county health officials, facility in-charges, program managers, and county finance department representatives to capture perspectives on feasibility, barriers, facilitators, and the sustainability of integration. In particular, the KIIs explored the perceptions and readiness of finance departments to domesticate and budget for STH/SCH activities within county health plans. FGDs engaged caregivers, CHPs, and other community members to provide community-level insights into acceptability, trust, and user experiences with integrated vs. campaign-based delivery. These insights informed recommendations on how to strengthen county-level planning, financing, and policy for the sustainable integration of NTD services into PHC. Participants were selected using purposive sampling that targeted healthcare workers, CHVs, caregivers, and teachers. Limited snowballing was used in identifying additional reliable stakeholders. Most of the interviews lasted 60–90 min while FGDs ran for 90 min depending on level of engagement from the participants. Consent was obtained before audio-recording the sessions and later transcribed verbatim for analysis. Thematic analysis was used in data analysis supported by SPSS version 29 to help in coding and theme development. To ensure there was rigor, we conducted double coding using two independent researchers who helped resolve discrepancies through discussion. Checking of members was done where selected respondents reviewed findings to confirm accuracy and careful examination of qualitative results. Thematic analysis was done for qualitative data.

### 2.5 Data quality assurance

The data quality was assured through various approaches. This included doing piloting and pre-testing of the tools. Pretesting of the tool was done in 1 day, after training the enumerators. Through training and piloting tools, the research team was able to identify and adjust potential issues arising from formulation of the questionnaires and redundancy.

To ensure high data quality, multiple control measures were instituted across all stages of data collection. The household questionnaire was programmed in KOBO Toolbox with integrated skip logic, constraints, validation checks, geotagging, and time stamping to enhance accuracy and minimize entry errors. Prior to the main survey, all tools were pre-tested to refine clarity, reliability, and usability.

For quantitative data, supervisors closely monitored submissions for completeness and accuracy. KOBO's real-time monitoring dashboard enabled immediate flagging and correction of inconsistencies during data collection. In cases where instant review was not feasible, daily synchronization of forms facilitated post-collection audits, allowing supervisors to generate error reports, cross-check entries with field notes, and provide timely feedback before subsequent data collection rounds.

For qualitative tools, deliberate steps were taken to reduce inter-observer variability. Research assistants participated in joint training sessions featuring role-plays and pilot testing of discussion guides to standardize techniques in probing, note-taking, and translation. Supervisors conducted spot checks during KIIs and FGDs to ensure consistent facilitation and adherence to protocols. Daily debrief sessions further enabled the team to compare notes, resolve discrepancies, and align on interpretation of responses.

Additionally, daily briefing meetings between the consultant and the Amref team were held to review field progress, identify emerging data quality concerns, and agree on corrective actions-thereby safeguarding the overall integrity and reliability of both qualitative and quantitative data throughout the study.

### 2.6 Ethical considerations

The study was approved in January 2025, by the University of Eastern Africa, Institutional Scientific and Ethic Review Board of BARATON (Ref: B1422192025). The clinical trial permission number was NCT07507461.

The survey team obtained informed consent from all participants before data collection. For children, they gave assent, and supported by consent from their parents and guardians. The assent and consent were given after proper introduction of the survey objectives, scope, and mentioning of the ethical considerations to them (respondents). Respondents were informed of their right to refuse participation or to withdraw at any stage without any consequence on the services they received. Information provided during interviews was not linked to any individual in the final report. All responses were treated confidentially and used solely for the purposes of this evaluation. Only general identifiers (such as geographical unit, gender, and age when reported voluntarily) were used. The researchers did not collect any information that could directly identify an individual was not collected or reported. Household-level GPS coordinates were anonymised in the final dataset by aggregating to ward level, ensuring individual households could not be identified while retaining spatial analysis capability. All team members had ethics training before study implementation, and only authorized members of the survey team had access to raw data.

## 3 Results

### 3.1 Socio-demographic characteristics

A total of 1,229 and 1,130 household interviews at baseline and end line respectively were done, out of a target of 1,016 exceeding the minimum target of 1,016 required for power for the power to detect significant differences between groups. There was an average decrease of 7.9% in households reached at endline, attributed to relocations and the observance of proportional sampling in clusters. In both the baseline and endline rounds, the distribution of participants between the two groups were statistically similar (as tested using chi-square test, baseline *p* = 0.758; endline *p* = 0.172) (see Table 1). This is also the case with gender distribution, where we observed a predominance of female respondents at 68% and 66% at baseline and endline, respectively. Age patterns were also statistically similar between the two arms for both rounds. At endline, the proportion of medium-sized households increased from 51% to 55%, while large households (>6 members) decreased from 31% to 24%. However, the household size between arms remained statistically similar (baseline *p* = 0.424; endline *p* = 0.099), confirming continued comparability. Statistically significant differences between arms were found in educational attainment and in the dominant livelihood source (see Table 1). There was higher secondary school attainment in intervention areas, and higher salaried employment in control areas at baseline.

**Table 1:** Comparison of socio-demographic characteristics of respondents at baseline and endline.

Characteristics	Baseline				Endline			
	Total (*N* = 1,229)	Control (*N* = 600)	Intervention (*N* = 629)	*p-values*	Total (*N* = 1,130)	Control (*N* = 559)	Intervention (*N* = 571)	*p-values*
County of residence								
Bungoma	385 (31%)	192 (50%)	193 (50%)	0.758	362 (32%)	184 (51%)	178 (49%)	0.172
Kakamega	410 (33%)	205 (50%)	205 (50%)		384 (34%)	200 (52%)	184 (48%)	
Transnzoia	210 (17%)	97 (46%)	113 (54%)		177 (16%)	75 (42%)	102 (58%)	
Vihiga	224 (18%)	106 (47%)	118 (53%)		207 (18%)	100 (48%)	107 (52%)	
Gender of the respondent								
Male	390 (32%)	185 (47%)	205 (53%)	0.508	380 (34%)	189 (50%)	191 (50%)	0.898
Female	839 (68%)	415 (49%)	424 (51%)		750 (66%)	370 (49%)	380 (51%)	
Age categories (Yrs)								
18–24	34 (3%)	17 (50%)	17 (50%)	0.395	51 (5%)	25 (49%)	26 (51%)	0.72
25–34	239 (19%)	111 (46%)	128 (54%)		205 (18%)	95 (46%)	110 (54%)	
35–44	283 (23%)	133 (47%)	150 (53%)		243 (22%)	121 (50%)	122 (50%)	
45–54	262 (21%)	140 (53%)	122 (47%)		224 (20%)	116 (52%)	108 (48%)	
55–64	184 (15%)	82 (45%)	102 (55%)		190 (17%)	88 (46%)	102 (54%)	
Above 64	227 (18%)	117 (52%)	110 (48%)		217 (19%)	114 (53%)	103 (47%)	
Household size								
1–3 people	222 (18%)	100 (45%)	122 (55%)	0.424	241 (21%)	109 (45%)	132 (55%)	0.099
4–6 people	628 (51%)	315 (50%)	313 (50%)		619 (55%)	324 (52%)	295 (48%)	
Above 6 people	379 (31%)	185 (49%)	194 (51%)		270 (24%)	126 (47%)	144 (53%)	
Level of education								
No formal education	337 (27%)	172 (51%)	165 (49%)	0.098	308 (27%)	156 (51%)	152 (49%)	0.022^*^
Primary school	520 (42%)	237 (46%)	283 (54%)		455 (40%)	202 (44%)	253 (56%)	
Secondary school	255 (21%)	138 (54%)	117 (46%)		277 (25%)	155 (56%)	122 (44%)	
University	117 (10%)	53 (45%)	64 (55%)		90 (8%)	46 (51%)	44 (49%)	
Main source of income								
Business	216 (18%)	109 (50%)	107 (50%)	0.145	205 (18%)	100 (49%)	105 (51%)	< 0.001^*^
Daily wage labor	295 (24%)	126 (43%)	169 (57%)		216 (19%)	94 (44%)	122 (56%)	
Farming	594 (48%)	300 (51%)	294 (49%)		587 (52%)	284 (48%)	303 (52%)	
Salaried job	87 (7%)	48 (55%)	39 (45%)		75 (7%)	55 (73%)	20 (27%)	
Others	37 (3%)	17 (46%)	20 (54%)		47 (4%)	26 (55%)	21 (45%)	

Significance levels: ^*^denotes p < 0.05.

### 3.2 Communication channels for STH/SCH deworming awareness

The study observed a shift in the main source of information on mass drug administration during the endline. At baseline, Community Health Promoters were the main source of information across the arms. By endline, dependence on CHPs as the primary source of information decreased from 48% at baseline to 17% at endline for the control groups. Respondents increasingly reported getting information through mass media (radio, loudspeaker announcements, posters, social media) and through community gatherings and public address systems. Health facilities also became more visible in the control arm, with 20%−27% of respondents across older age bands reporting facilities as a channel at endline compared to 15% at baseline (see Table 2).

**Table 2:** Summary of communication channels for STH/SCH deworming awareness.

Communication channel	Baseline - control (%)	Endline - Control (%)	Baseline - Intervention (%)	Endline - Intervention (%)
CHPs	48	17	40	20
Health facilities	15	24	16	30
Community meetings/Barazas	12	22	19	23
Mass media (Radio, TV, and social media)	16	26	19	24
Outreach clinics/Family & Social Networks	-	7	-	8
Schools	3	7	2	10
**Dominant mode**	CHPs	Mixed (Media + Baraza)	CHPs	Mixed (Facility + CHP + Media)

The awareness variable was derived from a binary yes/no question, coded as 1 for “yes” and 0 for “no” before logistic regression. Butsotso East Ward was chosen as the reference category due to its relatively high awareness level, while males were set as the reference group given their lower perceived awareness. Tertiary education was treated as the highest awareness category, and salaried employment was considered to reflect better awareness compared to other livelihood groups. Adjusted odds ratios were controlled for ward, age, gender, education, and household income.

As for the intervention arm, the role of health facilities became more prominent, with up to 30% of respondents in some age groups (notably 25–44 years) citing facilities as a source of information at endline. Mass media and community meetings also remained important, each accounting for 21%−25% among adults aged 25–54 years. In addition, channels that were not widely reported at baseline such as outreach clinics and information passed through relatives and social networks appeared at endline in intervention areas. Communication was increasingly delivered through routine service delivery touchpoints and informal face to face networks linked to those services as evidence by these quotes: “*When we go for the clinic, the nurse tells us about the deworming tablets. Sometimes they give them right there. We also hear during the health talks at the outpatient area*” (FGD, Community, Vihiga). A health facility head from an intervention site in Trans Nzoia added: “*Integration has helped because now deworming is talked about in ANC, PNC, and OPD. People hear about it every time they come to the facility, not only during campaign days.”*

In comparison, both the intervention and control groups experienced a decline in reliance on Community Health Promoters (CHPs) as the primary source of deworming information, accompanied by increased use of health facilities, mass media, community meetings, and social networks. Although health facilities appeared to play a more prominent role in intervention areas, similar communication shifts were observed in control areas, suggesting that the changes may reflect broader programmatic or contextual developments rather than PHC integration alone. Therefore, while PHC integration may have contributed to the diversification of communication channels, the evidence does not conclusively demonstrate a unique intervention effect.

### 3.3 Mass drug administration (MDA) awareness and uptake

A logistic regression model was done to examine ward and individual level predictors of MDA awareness and uptake following integration of STH/SCH treatment into PHC delivery structures. A binary variable of those aware of MDA and those not aware, controlling for demographic variables. This model adjusted for age, gender, education level, and household income to isolate the influence of programmatic and structural factors associated with implementation intensity and population engagement. Butsotso East Ward, which recorded very high participation during MDA campaigns, was used as the reference category for both baseline and endline surveys. At endline, the results revealed significant geographic heterogeneity in MDA awareness and uptake across wards. The adjusted odds ratios for residents in Busali, Marenyo-Shianda, Kisa East and South Kabras (control arm) showed that they were 80%−90% less likely to report awareness or participation compared to those in Butsotso East.

In contrast, Wards in the intervention arm such as Kwanza (AOR = 4.7, *p* = 0.172) and Matisi (AOR = 3.6, *p* = 0.265) showed elevated, though statistically non-significant, odds of MDA awareness, indicating improved awareness where PHC integration implemented (see Table 3). None of the socio-demographic variables that is, age (AOR = 1.00, *p* = 0.888), gender (AOR = 1.1, *p* = 0.681), education (AOR = 1.0, *p* = 0.963), or income (AOR = 0.9, *p* = 0.400), showed significant associations with MDA awareness or uptake. It is important to note that this endline finding mirrors the baseline analysis.

**Table 3:** Logistic regression of factors influencing MDA awareness and uptake at endline.

Study variable	Adjusted odds ratio (AOR)	95% confidence interval	*p-value*
Wards			
**Butsotso East Ward**	Ref		
Bokoli Ward	0.5	0.1–2.0	0.333
Busali Ward	0.1	0.0–0.2	**< 0.001**
Chesikaki Ward	0.6	0.1–2.3	0.419
East Wanga Ward	1.9	0.3–11.1	0.487
Kabula Ward	N/A		
Kamukuywa Ward	0.6	0.2–2.6	0.527
Kaptama Ward	0.6	0.1–2.4	0.460
Kisa East Ward	0.2	0.0–0.5	**0.004**
Kisa North Ward	0.3	0.1–1.4	0.136
Kwanza Ward	4.9	0.5–45.6	0.166
Marenyo-Shianda Ward	0.1	0.0–0.3	**< 0.001**
Matisi Ward	3.3	0.3–30.9	0.303
Matulo Ward	N/A		
Mayoni Ward	0.9	0.2–4.0	0.913
Muhudu Ward	0.9	0.2–3.9	0.893
Musanda Ward	N/A		
Namwela Ward	0.5	0.1–2.0	0.327
Sitatunga Ward	N/A	–	
South Bukusu Ward	0.6	0.2–2.6	0.525
South Kabras Ward	0.2	0.1–0.7	**0.012**
Tambua Ward	N/A		
Tuwani Ward	0.7	0.1–3.0	0.592
West Sabatia Ward	1	0.2–4.6	0.951
Age category			
18–24 yrs	0.5	0.2–1.4	0.172
**25–34 yrs**	Ref		
35–44 yrs	1.3	0.7–2.5	0.391
45–54 yrs	1.3	0.7–2.5	0.463
55–64 yrs	1.3	0.6–2.6	0.521
Above 64 yrs	0.9	0.4–1.8	0.710
Gender of the respondent			
Female	1.1	0.7–1.7	0.685
**Male**	Ref		
Education of the respondent			
No formal education	1.9	0.8–4.3	0.146
Primary school	1.7	0.8–3.7	0.177
Secondary school	3.8	1.6–9.0	**0.003**
**Tertiary school**	Ref		
Household income			
**Salaried**	Ref		
Business	1.5	0.6–4.1	0.416
Daily wage labor	1.6	0.5–4.5	0.408
Farming	1.5	0.6–4.0	0.369
Other (specify)	1.5	0.4–5.4	0.548
**Constant**	3.9	1.0–15.8	0.052

^*^p-values in bold show significance association

Comparison of baseline and endline regression results highlights a clear shift in the determinants of MDA awareness and uptake following PHC integration. At baseline, wards such as Kaptama, Kisa East, Marenyo-Shianda, Matulo, South Kabras, and West Sabatia exhibited significantly higher odds of awareness and participation than the reference ward, reflecting the strength of vertically managed, campaign-driven mobilization. By endline, however, several of these same wards (particularly Kisa East, Marenyo-Shianda, and South Kabras) recorded significantly lower odds, demonstrating that campaign related gains were not sustained once burst of activities subsided.

On the other hand, intervention linked wards under the integrated PHC model showed stable or improving awareness, reflecting the effect of PHC system reinforcement. Demographic predictors remained non-significant across both time points, confirming that health facilities systems and delivery models (campaign vs. integrated) were the primary determinants of awareness and uptake.

### 3.4 Delivery of STH/SCH treatment

The PHC-integrated package was designed to operate through two primary channels: facility-based delivery and community-based delivery. This entailed the embedding of deworming services into routine services in the Maternal and Child Health, and Outpatient department services in facilities, and working though the existing CHPs during their schedules household visits. This was in contrast to the control arm's campaign model which relied heavily on door-to-door drug administration by Community Drug Distributors (CDDs). Baseline data showed that access was strongly reliant on CHPs in both arms, with on 3.9% receiving deworming tablets from a health facility (see Table 4). At endline, we observed a significant increase of the proportion who received treatment from facilities. Arm-level comparison at endline shows that 90% of all respondents receiving deworming drugs from a heath facility were from intervention areas.

**Table 4:** Comparison of channels for receiving deworming drugs.

Source of treatment	Baseline *n* (%)	Endline *n* (%)	*p-value*
Health facility	48 (3.9)	71 (6.3)	0.008^*^
Household visits	1,173 (95.5)	1,031 (91.2)	< 0.001^*^
School	58 (4.7)	241 (21.3)	< 0.001^*^
Market	0 (0.0)	20 (1.8)	< 0.001^*^
Religious institutions	0 (0.0)	8 (0.7)	< 0.001^*^
Other venues	0 (0.0)	6 (0.5)	< 0.001^*^
Total respondents (*n*)	1,229	1,130	-

Significance levels: ^*^denotes p < 0.05.

A significant rise in the provision of treatments using facilities was seen in the intervention areas vs. the control areas, indicating that PHC integration successfully expanded the role of health facilities in deworming service delivery (see Table 4). However, community-based approaches continued to play an important role across both groups.

### 3.5 STH/SCH deworming program reach

Program reach reflects the proportion of surveyed individuals who reported being offered deworming drugs during the study period, regardless of whether they actually swallowed the tablets. When disaggregated by study arm, program reach remained high in both the control and intervention areas but showed a consistent advantage in control sites. In control sites, program reach was 92% (95% CI: 92.9–94.6). In intervention sites, program reach was 90% (95% CI: 91.5–93.4) (see Table 5). Both arms surpassed the WHO threshold of 85%, indicating good operational performance, but the control arm showed slightly higher reported offer of treatment compared to the intervention arm.

**Table 5:** Epidemiological coverage for STH/SCH deworming during the study period.

County	Ward	Total surveyed	Total treated	% epidemiological coverage	95% CI
Control					
Bungoma	Bokoli	274	257	94	90.2–96.1
Bungoma	Chesikaki	218	185	85	79.4–89.1
Bungoma	Kamukuywa	300	286	95	92.7–97.5
Bungoma	South Bukusu	265	243	92	87.7–94.5
Kakamega	Kisa East	184	164	89	83.7–92.9
Kakamega	Mayoni	244	211	86	81.5–90.2
Kakamega	Musanda	259	253	98	94.9–99.0
Kakamega	South Kabras	366	337	92	88.8–94.4
Trans Nzoia	Sitatunga	159	124	78	70.8–83.8
Trans Nzoia	Tuwani	191	148	77	71.0–82.9
Vihiga	Tambua	223	222	100	N/A
Vihiga	West Sabatia	228	204	89	84.7–92.9
**Sub total**		**2,911**	**2,634**	**90**	**89.4–91.6**
Intervention					
Bungoma	Kabula	301	274	91	87.2–93.8
Bungoma	Kaptama	239	212	89	84–92.2
Bungoma	Matulo	232	191	82	76.8–86.7
Bungoma	Namwela	269	259	96	93.2–98
Kakamega	Butsotso East	259	211	81	76.2–85.8
Kakamega	East Wanga	287	253	88	83.8–91.4
Kakamega	Kisa North	139	121	87	80.3–91.7
Kakamega	Marenyo-shianda	217	185	85	79.8–89.4
Trans Nzoia	Kwanza	237	232	98	95–99.1
Trans Nzoia	Matisi	249	196	79	73.2–83.4
Vihiga	Busali	278	257	92	88.7–95.0
Vihiga	Muhudu	286	265	93	89.0–95.2
**Sub total**		**2,993**	**2,656**	**89**	**87.6–89.8**
**Total**		**5,904**	**5,290**	**90**	**88.8–90.4**

The authors also observed that program reach was not only high among school-age children (5–14 years), but also among adults. Many wards had over 80% percent of the adult population offered the drug, suggesting that drug offers are no longer restricted to school distribution alone.

In both intervention and control areas, program reach was high, exceeding the WHO threshold of 85%. Although control areas reported slightly higher reach (92%) compared to intervention areas (90%), the difference was relatively small (see Table 5). These findings indicate that both delivery models were effective in reaching target populations, with no clear evidence of superiority for either approach.

### 3.6 Preventive chemotherapy coverage

Preventive chemotherapy reflects the proportion of the eligible population that actually swallowed the deworming tablets during the study period. According to the WHO CES guidance, a PC of above 75% is the minimum required for public health impact, while 85% is the minimum required for interruption of transmission and sustained elimination of the two diseases as public health problems.

Control areas recorded a slightly higher PC coverage of 92% (95% CI: 92.6–94.5), while intervention areas achieved 90% (95% CI: 90.7–92.7) (see Table 6). Although the difference is minor, it aligns with expectations during transition phases of integration, where control sites benefit from traditional, high-intensity campaign mobilization, whereas intervention sites rely on the evolving PHC infrastructure to deliver treatment through routine health facility visits, community outreach, and support from CHPs. Importantly, the overlapping 95% confidence intervals indicate no statistically significant difference between the two arms, underscoring that integration into PHC systems maintained effective coverage levels comparable to campaign-based delivery.

**Table 6:** Preventive chemotherapy coverage for STH/SCH deworming during the study period.

County	Ward	Total surveyed	Treated	% PC coverage	95% CI	% Cov. 1 years	% Cov. 2–4 years	% Cov. 5–14 years	% Cov. 15+ years	% Cov. Male	% coverage female
Control											
Bungoma	Bokoli	271	257	95	93.2–98.0	33	100	100	93	95	94
	Chesikaki	211	185	88	84.8–93.3	0	92	97	83	86	90
	Kamukuywa	296	286	97	95.5–99.1	100	100	100	94	97	97
	South Bukusu	261	243	93	91.9–97.3	83	100	100	89	95	91
Kakamega	Kisa East	181	164	91	89.6–96.9	60	100	100	87	88	93
	Mayoni	243	211	87	86.0–93.7	100	92	95	82	85	88
	Musanda	259	253	98	96.9–99.8	0	100	98	97	98	97
	South Kabras	358	337	94	93.4–97.6	100	100	99	90	95	93
Trans Nzoia	Sitatunga	157	124	79	75.1–87.5	20	100	61	86	78	80
	Tuwani	190	148	78	73.5–85.2	100	75	88	73	73	83
Vihiga	Tambua	222	222	100	N/A	100	100	100	100	100	100
	West Sabatia	228	204	89	87–94.5	50	93	90	90	96	84
**Sub total**		**2,877**	**2,634**	**92**	**92.6–4.5**	**74**	**96**	**96**	**89**	**92**	**91**
Intervention											
Bungoma	Kabula	299	274	92	91.5–96.9	100	100	98	87	90	93
	Kaptama	233	212	91	88.8–95.7	0	95	92	91	91	91
	Matulo	227	191	84	82.9–91.7	0	84	89	81	86	83
	Namwela	269	259	96	93.6–98.3	67	96	100	95	96	96
Kakamega	Butsotso East	256	211	82	78.6–87.8	50	100	89	79	80	85
	East Wanga	283	253	89	87–93.9	33	90	97	87	90	89
	Kisa North	136	121	89	83.9–94.3	33	50	100	89	92	87
	Marenyo-shianda	212	185	87	86.3–94.4	20	93	98	85	88	87
Trans Nzoia	Kwanza	237	232	98	95.0–99.1	100	89	100	98	98	98
	Matisi	249	196	79	73.5–83.7	50	86	85	75	77	80
Vihiga	Busali	278	257	92	93.6–98.2	43	100	100	90	93	92
	Muhudu	285	265	93	93.8–98.3	100	100	97	90	95	91
**Sub total**		**2,964**	**2,656**	**90**	**90.7–92.7**	**46**	**93**	**95**	**87**	**90**	**90**
**Total**		**5,841**	**5,290**	**91**	**92.0–93.3**	**62**	**94**	**96**	**88**	**91**	**90**

In both study arms, preventive chemotherapy coverage exceeded WHO-recommended targets, with control areas recording 92% and intervention areas recording 90% (see Table 6). The overlapping confidence intervals indicate that the observed difference was not statistically significant. Consequently, PHC integration appears to have maintained coverage levels comparable to the traditional campaign-based model rather than demonstrating a measurable improvement.

### 3.7 Compliance with STH/SCH deworming

This section is concerned with to the proportion of individuals who actually swallowed the deworming tablets among those who were offered them during the study period. It reflects not only the efficiency of drug administration but also the degree of community trust, acceptance, and adherence to treatment. During the June 2025 period, compliance was exceptionally high across both study arms, averaging 99% overall. Ward-level analysis confirm. All control wards (including Bokoli, Chesikaki, Kamukuywa, and South Bukusu in Bungoma; Kisa East, Mayoni, Musanda, and South Kabras in Kakamega; Sitatunga and Tuwani in Trans Nzoia; and Tambua and West Sabatia in Vihiga) recorded full compliance at 100% (see Table 6). Intervention wards displayed similarly strong adherence, with most reporting between 99% and 100%. The only minor deviations were Matulo Ward at 95% and Marenyo-Shianda Ward at 97%, which still reported high compliance (see Table 6). The minimal difference between the two arms demonstrates that integrating deworming into PHC has maintained the adherence traditionally achieved through campaigns.

Similarly, compliance rates were exceptionally high in both intervention and control groups, averaging approximately 99%. Most wards in both groups achieved compliance levels between 99% and 100%, indicating strong acceptance of treatment irrespective of the delivery approach. Given the minimal differences observed, the findings suggest that PHC integration maintained, rather than substantially improved, adherence to treatment.

### 3.8 Epidemiological coverage for STH/SCH deworming

Epidemiological coverage represents the proportion of the total population in the surveyed communities that actually received and swallowed the deworming tablets, regardless of eligibility category or prior offer. According to WHO CES guidance, epidemiological coverage above 65% is considered the minimum level required to achieve effective control of STH and SCH, while levels approaching or exceeding 85% are indicative of programs on course toward transmission elimination and sustainable public health impact. The overall epidemiological coverage across the study population was 90% (95% CI: 88.8–90.4), well above the global threshold for elimination level impact. In control areas, epidemiological coverage averaged 90% (95% CI: 89.4–91.6), slightly higher than in intervention areas, where coverage was 89% (95% CI: 87.6–89.8) (see Table 6).

When examined by age group, the highest epidemiological coverage was observed among school-aged children (5–14 years), who recorded 96% overall, followed by preschool children (2–4 years) at 94%. These outcomes reaffirm the strength of school and community-based platforms in delivering PC to child populations. Importantly, adults (15 years and above) also registered substantial coverage at approximately 88%, reflecting the expanding reach of PHC linked delivery into households and community health service points.

Male epidemiological coverage stood at 91% and female coverage at 90%, a difference that was not statistically significant (chi-square value, *p* > 0.05). This near parity in treatment across genders indicates that both delivery approaches effectively reached men and women alike, without any observable barriers influencing treatment uptake.

In comparison, epidemiological coverage remained high across both groups, with control areas achieving 90% and intervention areas achieving 89%. Coverage levels were similarly high across age groups and genders, and no substantial disparities were observed between study arms. These results suggest that both delivery approaches were successful in achieving effective population-level coverage, with limited evidence of a distinct advantage associated with PHC integration.

### 3.9 Effectiveness of STH/SCH deworming

Effectiveness in the context of this study refers to the extent to which the deworming intervention achieved its intended coverage outcomes across study arms and counties during the study period. It integrates program reach, compliance, and PC coverage to assess how well the delivery platforms-whether campaign-based or integrated into PHC-performed in ensuring that eligible individuals were not only reached but also treated. According to WHO guidelines, a PC coverage rate of at least 75% is the global benchmark for evaluating the effectiveness of deworming campaigns.

Control areas achieved slightly higher performance, with PC coverage at 92% and epidemiological coverage at 90%, compared to 90% and 89%, respectively in intervention areas (see Table 7). Across the study counties, the deworming programme achieved outstanding effectiveness, with all major indicators far exceeding the WHO 75% benchmark. Program reach stood at 91% (95% CI: 92.5–93.8), indicating that almost all eligible individuals were offered treatment. Compliance was exceptionally high at 99%, demonstrating high willingness to swallow the tablets once offered. PC coverage reached 91% (95% CI: 92.0–93.3), while epidemiological coverage stood at 90% (95% CI: 88.8–90.4) (see Table 7). This shows that both arms achieved the levels of effectiveness necessary for successful mass drug administration. Despite reduced reliance on mass mobilization, PHC linked areas maintained equally high effectiveness, confirming that integration can achieve comparable results through more sustainable and health system driven processes.

**Table 7:** Effectiveness of deworming delivery during the study period.

County	Ward	PC coverage (%)	95% CI	Effectiveness of MDA for STH
Bungoma	Bokoli	95	93.2–98.0	Highly effective
	Chesikaki	88	84.8–93.3	Effective
	Kamukuywa	97	95.5–99.1	Highly effective
	South Bukusu	93	91.9–97.3	Highly effective
Kakamega	Kisa East	91	89.6–96.9	Highly effective
	Mayoni	87	86.0–93.7	Effective
	Musanda	98	96.9–99.8	Highly effective
	South Kabras	94	93.4–97.6	Highly effective
Trans Nzoia	Sitatunga	79	75.1–87.5	Effective
	Tuwani	78	73.5–85.2	Moderately effective
Vihiga	Tambua	100	N/A	Highly effective
	West Sabatia	89	87.0–94.5	Highly effective
Overall–control arm	–	92	92.6–94.5	Highly effective
Overall–intervention arm	–	90	90.7–92.7	Effective
Combined average	–	91	92.0–93.3	Highly effective

Both intervention and control groups achieved effectiveness indicators that substantially exceeded WHO benchmarks for deworming programs. Although control areas reported marginally higher preventive chemotherapy and epidemiological coverage, the differences were small and unlikely to represent meaningful programmatic advantages. The findings therefore suggest that PHC-integrated delivery can achieve effectiveness levels comparable to those of the conventional campaign-based approach, but they do not provide strong evidence that integration results in superior outcomes.

### 3.10 “Never Treated” households

From the two groups, intervention at 1,229 and control at 1,130, (total of 2,359) there were 5% of households (*n* = 118) reported never having been treated for deworming. Across the combined sample, the most common reason cited for non-treatment was ineligibility for treatment (56%), followed by absence during the deworming period (17%), no visit by a CDD/CHP (11%), and other unspecified factors such as illness or migration (14%) (see Table 8). Only 1% of respondents attributed non-treatment to a lack of awareness, confirming that information diffusion about deworming was nearly universal in the intervention group/arm. One of the key findings in the report was that only 5% of households never treated, primarily due to ineligibility (56%) or absence (17%) and not lack of awareness. This was significantly lower compared to about a quarter reported in the 2024 MDA Coverage Evaluation Survey (CES), potentially reflecting the impact of the integrated approach (see Table 8).

**Table 8:** Reason for never receiving deworming treatment.

Reason for non–treatment	Control (*n* = 143)	Intervention (*n* = 160)	Combined (*n* = 303)
Not present during campaign	17%	18%	17%
Lack of awareness	1%	2%	1%
No visit by CDD/CHP	13%	9%	11%
Not eligible for treatment	56%	57%	56%
Other reasons (illness, relocation, refusal)	13%	14%	14%

Differences between arms were not statistically significant (p > 0.05).

In the control arm, 143 respondents (47% of all untreated households) reported never having been treated. Ineligibility was the most frequently cited reason because of absence during school-based distribution, age beyond the target group, or not considered at risk. A small proportion (13%) mentioned other contextual reasons such as illness, refusal, or unclear eligibility status. Only one respondent (1%) attributed their exclusion to a lack of awareness. In the intervention arm, 160 respondents (53% of all untreated households) were recorded as never treated. The distribution of reasons mirrored that of the control arm: 57% cited ineligibility, 18% absence during the campaign, 9% lack of a visit by a CHP/CDD, and 14% other reasons such as migration or relocation. Awareness-related exclusion was also minimal in this arm (2%). The similarity of these proportions across arms indicates that the PHC-integrated model did not face additional information barriers compared to the MDA campaign model.

However, ward-level analysis revealed localized implementation gaps that help explain small variations in coverage. In both arms, the majority of “never treated” cases clustered in specific wards with unique operational or demographic characteristics. In the control arm, Tuwani (26 cases) and South Kabras (23 cases) accounted for a large share of untreated households. The reasons given included population mobility and dispersed settlement patterns. Similarly, in the intervention arm, Kabula (18 cases), Muhudu (19 cases), and Busali (17 cases) contributed highly to the total, suggesting that even under the PHC integrated model, rural wards with broad catchment areas faced outreach limitations.

Comparing the control and intervention groups, the proportion of households that had never received deworming treatment was low and relatively similar across the two groups. In both groups, non-treatment was primarily attributed to ineligibility, absence during treatment periods, and operational challenges rather than lack of awareness. The similarity in reasons reported across study arms suggests that PHC integration did not substantially alter the factors associated with non-treatment, although it may have contributed to maintaining low levels of exclusion.

## 4 Discussion

This study aimed to evaluate the outcomes of integrating MDA within PHC in Western Kenya. Literature shows a scarcity of studies investigating this strategy of NTD control, a critical review only identified 11 studies which were all published before 2003 (7). This could be because of the emphasis on MDA as a control strategy which is cost-effective and feasible in low resource settings (15). This work adds some evidence to the feasibility and efficacy of integration for the achievement of SDG 3.3 through Universal Health Coverage. The results of the study are promising and call for more investigations into the performance of the integration over a longer period. Results showed increased involvement of the primary healthcare system in various aspects deworming drug distribution after the implementation of the intervention.

### 4.1 On demographic characteristics

Baseline and endline populations were similar across intervention and control arms, without statistically significant differences for key demographic characteristics, including age, gender, and household size. This similarity enhances internal validity and provides support for causal interpretations of program impacts. The same methodological rigor has been applied to NTD program evaluations to produce balanced study groups that help researchers determine whether intervention outcomes were caused by interventions or confounding variables (4, 9, 16). In accordance with patterns found in community-based health research, most respondents were women. Women are frequently the primary caregivers and are also more likely to be involved in health-related surveys (17, 18). However, the differences in education and source of livelihood between arms reflect the underlying economic diversity in the participant groups; socio-economic diversity has been cited as one of the determinants of access to health services in NTD-endemic settings (10, 19). The observed findings also indicate that although demographic comparability was accomplished, socio-economic differences in context may still affect the uptake of interventions.

### 4.2 Communication channels

Transitioning from Community Health Promoters (CHPs) to other forms of communication (such as mass media or health facilities) represents a movement toward a more integrated health communication system. Studies have shown that integrating messages about neglected tropical diseases (NTDs) into routine health services improves sustainability (10, 14, 16). The growing use of health facilities for communication purposes also parallels findings from integrated programming in Mali and the Dominican Republic were continuing to reach people through facility-based communication has improved their knowledge of MDA beyond the campaign period (17, 18). In addition, the development of informal networks and social diffusion pathways further demonstrates previous conclusions about using community-based information ecosystems to support sustained awareness of MDA (7, 8). However, compared to the previous studies that identified CHPs as an important part of community mobilization (20), anticipating the shift of CHPs away from being the prime (but not sole) means of communicating messages, integration may change the traditional roles of communication.

### 4.3 MDA awareness and uptake

The research indicates that the primary factors affecting MDA awareness and uptake were programmatic/structural (particularly delivery models) vs. socio-demographic. Numerous sources support the assertion that health system design and implementation intensity are major determinants of successful NTD interventions (4, 9, 16) and that the observed drop in awareness in the post-campaign control wards could be attributed to the fact that vertical campaigns produce only temporary benefits, which cannot be sustained without system-level integration (8, 19). The stability of the intervention wards aligns with the literature that found integration into PHC systems results in improved continuity and resilience of service delivery (14, 15). Finally, the lack of significance for demographic variables further supports findings from global studies on NTDs that indicate program design is a more important contributor to uptake than individual-level factors (6).

### 4.4 On delivery of STH/SCH

As interventions evolved, the focus of drug distribution shifted from community-based systems to delivery through health facilities (i.e., within the overall framework of the District Health System). This shift is an important indicator of how PHC integration has changed the way service providers operate in their respective geographical areas. Consistent with existing literature that shows that integrating interventions for the treatment of NTDs into standard care increases accessibility to services via multiple points of contact (14, 17, 18), there has been a dramatic increase in the number of deliveries occurring at health facilities, particularly compared to earlier models that emphasized using community campaigns as the primary method of drug distribution (7, 8). While community-based programmes have historically achieved high coverage rates, evidence supports that the integration of NTD service delivery into a PHC system leads to improved sustainability and a reduction in programmatic fragmentation (10, 16). However, some studies suggest that there may be a negative impact on the ability to reach hard-to-reach populations due to an over-reliance on facility-based delivery systems if adequate outreach mechanisms are not maintained (19), underscoring the need for hybrid delivery models.

### 4.5 STH/SCH deworming program reach

Both arms of the program surpassed World Health Organization (WHO) standards for reach, with a higher number of people reached in the control areas, highlighting that mass drug administration campaigns have been effective at generating high coverage rates in a rapid manner (7–9). The slightly lower coverage in intervention wards hat stood at 90% to 92% happened due to various operational challenges such as delays in implementation. Other challenges included health worker workload gained from added integration tasks, variability in community mobilization effectiveness, and temporary drug-stock-outs during the transition phase. These challenges also affected uptake despite overall improvements in the system.

Additionally, while both arms had similar levels of reach due to the success of integrated primary health care (PHC) approaches, the higher reach rates in the intervention areas suggest that integrated PHC strategies are capable of generating similar levels of reach as have been documented in an intervention program conducted by the integrated neglected tropical disease (NTD) programs in Africa (14, 17). Furthermore, the inclusion of adults in the treatment coverage illustrates a movement toward including all members of a community in the provision of services, consistent with the WHO's roadmap which indicates an increasing emphasis on providing a broader range of populations with services than has previously been provided through school-based programs (6, 9), thus implying that integration may serve to increase equity in the provision of services.

### 4.6 Preventive chemotherapy

Coverage for preventive chemotherapy (PC) for both intervention and control arms maintained above WHO standard of 75%, with no statistically significant differences between the arms. The finding reflects evidence suggesting that high treatment coverage can be achieved using integrated systems similar to traditional campaigns, which provides additional support for integrated delivery systems (5, 14, 15). The control areas had a little more than the average for the campaigns, likely due to the high intensity of the campaign-based strategies as previously documented (7, 8). However, similar outcomes in the intervention areas provide additional rationale for the integration of integrated delivery systems into public health care (PHC) systems can provide sustained effective coverage while increasing the efficiency and viability of the programs (10, 16). This further supports the global movement toward integrated control strategies for NTDs.

### 4.7 Compliance with STH/SCH deworming

High levels of follow-through (compliance) seen in both groups tell us that the community supports the program. This corresponds to other NTD's programs showing that when people are offered drugs, they will take them (8, 9, 17). Almost everyone who received deworming did so because they had access to it; this has also been documented in all studies (7, 16). The consistent high follow-through with the integrated model supports that integration has not hindered community trust and/or acceptance (14). Lastly, it emphasizes how important it is to have drugs available for continuous access to maintain high levels of treatment usage.

### 4.8 Epidemiological coverage

Both of the delivery models demonstrated strong performance with regard to achieving the expected epidemiological coverage thresholds set forth by WHO; thus, the findings from both types of delivery systems provide some evidence to support this conclusion. Furthermore, this supports earlier evidence that suggests that both integrated and campaign approaches to delivering a drug will lead to high levels of geographic coverage (6, 7). In addition, children continue to have comparatively higher rates of geographic coverage in comparison to adults; therefore, it is likely that there will be continued success with using school-based and community-based platforms to deliver drugs (9, 11). However, although the adult population appears to be attaining significant levels of geographic coverage (14, 15), this suggests that the integration of PHC into drug delivery programs may provide an additional opportunity for expanding the availability of services beyond the traditional target populations. Furthermore, the gender parity observed among the populations supported the findings from other studies that indicated that properly designed programs can lead to equitable access to services throughout the population (10).

### 4.9 Effectiveness of STH/SCH deworming

The total effectiveness for the de-worming programme, when combining indicators, was high for both arms and exceeded the global benchmarks. This is consistent with literature demonstrating that both vertical and integrated approaches have similarly high levels of effectiveness when implemented as designed (5, 7, 8). The comparable effectiveness of the integrated approach enforces arguments for moving to health-system based delivery to ensure sustainability (14, 16). However, the control area's slightly higher performance reflects the sustained efficiencies from intensive campaigns, especially in the short term (19). These findings illustrate the need to balance short-term coverage gains with long-term systems strengthening.

### 4.10 “Never treated” households

The fact that very few households have never received treatment and that awareness is not a major barrier to access shows how well information has been shared between arms. These observations are consistent with findings from other MDA programs that have shown awareness is rarely a significant barrier in mature programs (8, 9, 17). On the other hand, structural factors (mobility, ineligibility, access to services, etc.) are the dominant barriers to utilization based on this study and previous studies on NTDs (16, 19). Since both arms have similar levels of untreated cases, we can conclude that integrating service delivery will not add new barriers for users, which is consistent with evidence on previous integrated programming (14, 15). However, the concentration of untreated cases in the same wards indicates that significant geographic disparities still exist, which has been documented in other studies reporting on hard-to-reach populations in high-performing programs (6, 11).

## 5 Study limitations and potential bias

Several limitations should be considered when interpreting these findings. First, the intervention period was only 3 months, which is insufficient to assess the long-term sustainability of PHC integrated deworming or to observe potential prevalence resurgence. Second, while wards were randomly assigned, the randomization was constrained by programmatic feasibility, introducing potential selection bias; baseline differences in education and livelihood sources between arms support this concern. Third, treatment coverage and compliance relied on self-reported household survey data, which may be subject to social desirability and recall bias, despite efforts to triangulate with facility records. Fourth, attrition of 7.9% between baseline and endline, attributed to household relocations and proportional sampling adjustments, may have introduced non-response bias if mobile populations differ systematically from settled households. Fifth, we measured coverage and awareness but did not directly measure infection prevalence post-intervention; therefore, we cannot confirm that integrated delivery achieves equivalent epidemiological impact. Finally, the study was conducted in four counties in Western Kenya with established MDA history; findings may not generalize to low-coverage settings or regions with weaker health systems. Future research should include longer follow-up periods (≥12 months), direct measurement of infection prevalence and cost-effectiveness analyses. The quasi-experimental design also limits one's ability to design strong causal inference, since wards were not randomly assigned to control and intervention groups. Hence, this increased the risk of selection bias and some unmeasured confounding issues that influenced outcomes. Also, even though deliberate efforts were done to ensure there was compatibility between wards, differences in baseline health system capacity, geographic accessibility, and community levels of engagement may have impacted the effectiveness of implementation. Also, there is possibility of implementation variability across wards. This is because the integration of deworming into primary healthcare relied on the capacity of workers. Reliability in supply chain, and supervision intensity. These contextual differences may have impacted outcomes independently of the intervention itself. The reliance on routine health records and self-reported data introduces potential reporting and bias for recall. As a result, this may overestimate or underestimate actual deworming coverage. Even though follow-up was done within the first 6 months, this period may be inadequate to capture long-term sustainability, patterns of reinfection, and change in behaviors. Also, issues such as migration, household mobility, and the lack of temporary absence of some respondents contributed to attrition. As a result, this might introduce non-response bias despite having relatively higher rates of retention. Furthermore, even as qualitative and quantitative methods we utilized, findings integration was limited. Hence, this lowered the depth of triangulation and explanation of observed differences. Future studies should therefore consider longer follow-up period, stronger integration of mixed methods, making randomized designs feasible, and making more robust monitoring of implementation fidelity in a bid to strengthen evidence on the effectiveness of integrated deworming initiatives in PHC systems.

## 6 Conclusion

The findings show that employing STH/SCH deworming with primary health care (PHC) systems can produce health outcomes similar to or better than those obtained from traditional model-based campaign approaches.

While the control areas had slightly higher program reach and preventive chemotherapy coverage than the intervention areas because they used intensive campaign approaches to deliver programs, the intervention areas showed stable or increasing awareness and better communication distribution through a variety of channels as well as greater integration of services into routine health facilities. Importantly, adherence to the protocols for all models was virtually the same; however, there were many examples where the epidemiological coverage obtained in both models exceeded the global standards for effective epidemiological coverage, demonstrating that strength of proven impact on the population level.

Additionally, structural and systemic factors (especially delivery channels and the degree of integration of services) were found to be greater contributors to the success of programs than any of the individual demographic factors associated with the population served. Nevertheless, it was also discovered that in all models, localized and “untreated” areas exist, suggesting that significant operational and contextual barriers still persist which need to be targeted and addressed. The performance of PHC-integrated SCH/STH treatment was found to be similar to those obtained through the traditional method.

### 6.1 Recommendations

Enhancing the sustainability and equity of deworming programs requires improvements in PHC-integrated delivery systems while maintaining the strategic features of high-intensity campaigns in remote areas. Investments should focus on strengthening health facility-based service delivery by integrating deworming indicators into facility-level performance dashboards, expanding networks for community health promoters, and using multiple forms of communication (for instance, mass media, social media) to reach the population. Targeted interventions should focus on addressing specific gaps in service coverage and outreach in population groups that move frequently or are located far apart from one another. In addition, ongoing monitoring and data-driven planning and flexible implementation strategies might help maintain high levels of coverage and compliance. Strengthening coordination among community and facility platforms, and integrating deworming into other health services (for instance, in maternal and child health) might improve the overall efficiency, resilience, and long-term impact of deworming programs.

We recommend that Kenya's MOH transitions deworming to PHC platforms in stable endemic areas, while retaining campaign-style outreach for highly mobile populations and low-coverage wards.

## Data Availability

The raw data supporting the conclusions of this article will be made available by the authors, without undue reservation.
